# Effects of *Debaryomyces hansenii* treatment on intestinal mucosa microecology in mice with antibiotic-associated diarrhea

**DOI:** 10.1371/journal.pone.0224730

**Published:** 2019-11-14

**Authors:** Ao Zeng, Maijiao Peng, Huizhi Liu, Zhaohui Guo, Jun Xu, Shengping Wang, Lu He, Zhoujin Tan

**Affiliations:** 1 Hunan Institute of Microbiology, Changsha, Hunan Province, China; 2 Hunan University of Chinese Medicine, Changsha, Hunan Province, China; University of Illinois at Urbana-Champaign, UNITED STATES

## Abstract

**Aim:**

To confirm the effects of *Debaryomyces hansenii* on intestinal microecology in mice with antibiotic-associated diarrhea (AAD).

**Methods:**

This study took the mucosal microecology as the entry point and an antibiotic mixture was used to induce diarrhea in mice. *D*. *hansenii* suspension was used to treat the mice and the bacterial communities of mucosa was analyzed using high-throughput sequencing.

**Results:**

The Shannon-Wiener index indicated that the sequencing depth is reasonable and reflected the majority of microbial information. The principal coordinate analysis results showed that mice in the treatment group and the normal group had a similar microbial community structure, while differences in microbial community structure were observed between the model group and the treatment group. The inter-group bacterial structures were analyzed at the phylum level and genus level. The results revealed that antibiotic treatment increased the proportion of *Proteobacteria* and decreased the proportion of *Bacteroides*, while *D*. *hansenii* treatment inhibited the increase in *Proteobacteria*. Linear discriminant analysis coupled with effect size measurements (LEfSe) suggested d that the beneficial bacteria *Candidatus Arthromitus* were the only common bacteria in the normal group (*P*<0.05).

**Conclusion:**

The treatment with *D*.*hansenii* could contribute to the maintenance of the structure of the mucosal microbiota in comparison with the normal group and inhibit the proliferation of opportunistic bacteria. However, high-dose antibiotic treatment causes mucosal dysbiosis and the proliferation of opportunistic bacteria during the self-recovery period, such as *Pseudoalteromonas*, *Alteromonas*, *Vibrio*.

## Introduction

Antibiotic-associated diarrhea (AAD) is a common adverse drug reaction with a probability of 5% to 35%[1]. The pathogenesis of AAD is complicated and has not yet been fully understood. We studied the mechanism of AAD using gut microbiota and mucosa, and found that the use of antibiotics can damage the intestinal physiology and microbial community structure, resulting in gut dysbiosis [2–4]. the direct stimulation of antibiotics on mucosa and gut dysbiosis aggravates intestinal mucosa damage and causes diarrhea[5–7]. Therefore, adjusting the intestinal microbiota and repairing the intestinal mucosal barrier can be effective means to treat AAD.

The gut microbiota is the largest and most complex microecological system in the human body. A normal and balanced intestinal microbiota plays an important role in resisting infection from pathogenic microorganisms and ensuring healthy growth conditions for animals. There are differences between intestinal mucosal microbiota and intestinal lumen microbiota. Compared with the intestinal lumen microbiota, the mucosal microbiota is closer to the host epithelium, and it has been proved that mucosal microbiota, *Lactobacillus*, *Bifidobacterium*, *Akkermansia muciniphila*, and *Candidatus arthromitus* all have mucosa adhesion ability. Therefore, in comparison with the intestinal lumen microbiota, the mucosal microbiota may play a more important role in the occurrence and development of gastrointestinal diseases[8].

At present, many strains of probiotics, such as *Bifidobacterium*, *Lactobacillus* and *Bacillus* SPP., have been widely used as microecological regulators for the treatment of dysbiosis diarrhea, but few applications of yeast have been reported. Actually intestinal yeast can not only stimulate the growth of probiotics, but also directly participate in the composition of biological barriers. A yeast strain isolated from mouse gut by our research team, was identified as *D*. *hansenii* after physiological, biochemical and molecular examinations [The sequence accession number in GenBank is KC534843(18S rDNA) and KC534843(26S rDNA)]. Its survival was determined in an acidic, cholestatic environment and artificial gastric fluid, which was considered to be in accordance with the conditions of the new microecological preparations. In addition, the therapeutic and intestinal microecology effects of dysbiosis diarrhea in mice were evaluated from the perspective of intestinal contents, and it was confirmed that *D*. *hansenii* has the ability to treat antibiotic-associated diarrhea and adjust the disordered intestinal microecology[9,10]. In this study, therapeutics effects of yeast on intestinal microecology and dysbiosis diarrhea were evaluated using high-throughput sequencing technology, to provide further evidence for the treatment of antibiotic-associated diarrhea with *D*. *hansenii* and theoretical guidance for the clinical application of microbiological preparations.

## Materials and methods

### Reagents

Reagents used in animal modeling included: Cefradine capsules (batch number: 160621) purchased from Suzhou zhong-hua Chemical and Pharmaceutical Industrial Co., Ltd. and Gentamicin sulfate injection (batch number: 5161111) purchased from Yichang Human well Pharmaceutical Co., Ltd., Reagents. The reagents used for DNA extraction included: Lysozyme, proteinase K, dNTP, *Taq* polymerase, acetate alcohol, acetone, *TE* buffer, Tris-saturated phenol-chloroform-isoamyl alcohol (25:24:1), 10×*Taq* buffer and Chloroform-isoamyl alcohol (24:1) purchased from Beijing Dingguo Changsheng Biotechnology Co., Ltd. Solutions such as 10% SDS, PBS, 3 mol/L sodium acetate, 5 mol/L NaCl, CTAB/NaCl, Tris, 0.5 mol/L EDTA and 70% ethanol were prepared in the laboratory.

Preparation of antibiotic mixture: the mouse model of AAD was established by our research team and the mice were administered a high concentration (62.5 g/L) of antibiotic mixture composed of gentamycin sulfate and cefradine in sterile PBS.

Preparation of *D*. *hansenii* suspension: The *D*. *hansenii* colony presented in white milky color and oval shape. Through microscopic observation, its cell presented in oval shape, and the asexual reproduction was gemmation. The yeast could ferment glucose, sucrose and D–mannitol, as well as assimilate peptone and potassium nitrate, while, it could not tolerate high concentration of alcohol, and the urease test was negative. The strain was cultured at 28°C on PDA medium for 3 days, then the *D*. *hansenii* strain was inoculated into a 500 mL Erlenmeyer flask containing 200 mL liquid PDA medium and shaken for 36 h at 28°C. The cells were collected by centrifugation at 2000×*g* for 4 min and then washed 1–2 times using the appropriate amount of stroke-physiological saline solution. The appropriate amount of sterile stroke-physiological saline solution was added to the cells. After the cells were counted using a hemocytometer, they were diluted to 10^10^ cells/mL and stored at 4°C for use[10].

### Animal grouping and mucosal acquisition

This study was carried out in strict accordance with the recommendations in the Guide for the Care and Use of Laboratory Animals of the National Institutes of Health. All procedures involving animals were reviewed and approved by the Animal Ethics and Welfare Committee of Hunan University of Chinese Medicine, Animal License SCXK (Xiang) 2013–0004. After 2 days of adaptive feeding, the mice were randomly divided into three groups (half male and half female): the normal group (MN), the model group (MM) and the treatment group (MD). The mice in the model group and the treatment group were given 0.35 mL antibiotic mixture composed of gentamycin sulfate and cefradine (23.33 mL/kg/d) by gavage, twice a day for 5 days. Correspondingly, the mice in the control group were given 0.35 mL sterile water by gavage, twice a day for 5 days. When symptoms (reduced activity, curled up, arched back, trembling and wet feces) were observed, this indicated that the AAD model was successfully established. On the sixth day, the mice in the treatment group were given 0.35 mL *D*. *hansenii* suspension by gavage, twice a day for 4 days, while the mice in the control group and model group were given 0.35 mL sterile water by gavage, twice a day for 4 days.

Following treatment, the mice were sacrificed by cervical dislocation, the intestinal mucosa in each group was collected according to the sterile intestinal mucosa collection method established by our research group[10]. Under sterile conditions, the jejunum was removed, and the contents of the intestinal wall were washed with sterile saline, and the intestinal mucosa was then scraped off using coverslips and preserved in a sterile centrifuge tube.

### Extraction of DNA

2 g mucosal samples preserved at 4°C were used for mucosal microbial DNA extraction. Firstly, the mucosal microorganisms were suspended by centrifugal oscillation, 5 mL of 0.1 mol/L phosphate buffered solution (PBS) was added to each tube, and then centrifuged at 200×*g* for 2 min. After washing twice with PBS, the supernatant was transferred into new sterile tubes and centrifuged at 10,000×g for 8 min. The cell pellets were collected, washed once with PBS, twice with acetone, and three times with PBS, and finally resuspended in 4 mL TE buffer. The above process was the sample pretreatment step.

DNA was extracted following the mouse intestinal DNA extraction method established by our research group [11]. In brief, 500 μL of the pretreated bacterial suspension was placed in a 1.5 mL sterile centrifuge tube, and added with 45 μL of TE buffer, 5 μL of proteinase K and 20 μL of lysozyme. The samples were incubated at 37°C for 30 min, mixed with 30 μL 10% SDS and allowed to react for 40 min at 37°C. The samples were shaken once every 10 min. Afterwards 80 μL of CTAB/NaCl and 100 μL 5 mol/L NaCI were added, thoroughly mixed, and the mixture was reacted at 65°C for 10 min. The DNA was extracted using the Phenol: Chloroform: Isoamyl Alcohol (25:24:1) extraction method.

### PCR amplification of the 16SrDNA V3+V4 variable region and preparation of sequencing library

The V3+V4 variable region common primer was synthesized by Shanghai Personal Biotechnology Co., Ltd. Primer sequence was 338F: (5’-ACTCCTACGGGAGGCAGCA-3’) and 806R: (5’-GGACTACHVGGGTWTCTAAT-3’). The PCR mixture (20 μL) contained 2 μL 10×Buffer, 2 μL 2.5 mmol/L dNTPs, 0.8 μL Forward Primer (5 μmol/L), 0.8 μL Reverse Primer (5 μmol/L), 2 μL Taq Polymerase, 0.2 μL BSA and 10 ng Template DNA. The PCR initiation denaturation temperature was 95°C (3 min), and cycling parameters were as follows: denaturation at 95°C (30 s), annealing at 55°C (30 s) and extension at 72°C (45 s), repeated for 29 cycles, with a final extension at 72°C (10 min). PCR products were detected by 2% agarose gel electrophoresis. In addition, the target segments were excised from the gel and purified using the gel recovery kit of AXYGEN company. Referring to the preliminary quantitative results of electrophoresis, the recovered products of PCR amplification were quantified by fluorescence. The fluorescence reagent was quant-it PicoGreen dsDNA Assay Kit, and the quantitative instrument was Microplate reader (BioTek, FLx800). The recovered concentration of all samples was not lower than 0.5ng/μl.

The TruSeq Nano DNA LT Library Prep Kit from Illumina was used to prepare the sequencing Library. Firstly, repair the end of the sequence of the amplification products mentioned above. Then the DNA fragment was amplified by PCR to enrich the sequencing library template. Eventually, the final fragment selection and purification of the library were performed by 2% agarose gel electrophoresis. Before the sequencing, the quant-it PicoGreen dsDNA Assay Kit was applied to quantify the library with the Promega QuantiFluor fluorescence Assay Kit. Library concentration was above 2 nM. The amplified V3+V4 variable region of 16SrDNA was subsequently sequenced by the Illumina MiSeq platform (Frasergen Co., Ltd).

### Analysis process and statistical analysis

First of all, the original data of high-throughput sequencing were screened according to the sequence quality[12]. Secondly, The sequences of the initial mass screening were identified and allocated to the corresponding samples according to the barcode information and primers, and the doubtful sequences such as chimera were removed[13]. Then, the sequences obtained above were merged and clustered into Operational Taxonomic Units (OTUs) with 97% sequence similarity, and the sequence with the highest abundance in each OTU was selected as the representative sequence of the OTU[13]. Afterwards, according to the abundance distribution of OTU in different samples, the diversity level of each sample was evaluated, and the sparse curve reflects whether the sequencing depth is reasonable. Subsequently, the specific composition of each sample (group) at different classification levels was analyzed, to check whether there was a statistical difference between the groups. SPSS 21.0 software (IBM Corp, Armonk, NY, USA) was used to analyze the measurement data. The pairwise samples t test was used to compare the statistical significance of differences, and P<0.05 or P<0.01 was considered statistically significant. Eventually, LEfSe analysis was done by submitting the relative abundance matrix at the genus level through the Galaxy online analysis platform, and the results were visualized. The spatial distribution characteristics of community samples based on microorganism phylogenetic relationship were acquired by Principal Coordinate Analysis (PCoA) of Weighted UniFrac Distances[14,15].

## Results

### The apparent characteristics of mice

The initiation of diarrhea in mice required 5 days of antibiotic treatment, and the *D*.*hansenii* treatment lasted 4 days[10]. The wetness of mice feces during study period was observed, and the results were shown in Table 1. Treatment with *D*.*hansenii* alleviated the symptoms of diarrhea in mice.

**Table 1:** The influence of the clinical features of mice.

	The dilution of feces after 5 days of molding	The dilution of feces after 4 days of treatment
MN	++	++
MD	−−	+
MM	−−	-

Note: +: degree of fecal dryness; -: degree of fecal wetness. The more minus signs, the higher the wetness

### Shared OTU analysis

**Fig 1:**
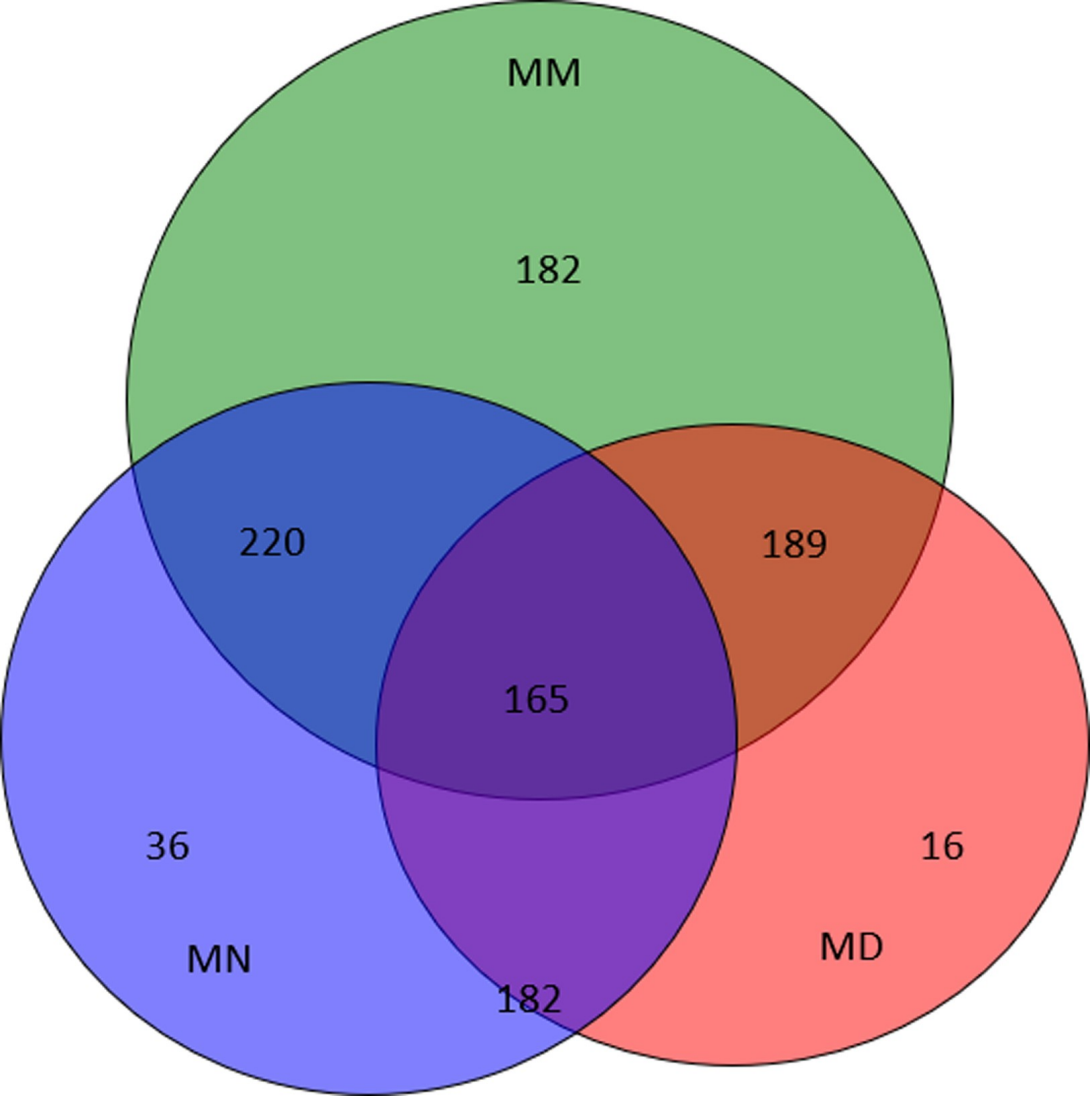
Venn diagram. Venn diagram of shared OTUs based on the sequences with over 97% similarity. The normal group (MN), the model group (MM) and the treatment group (MD).

When the sequencing was complete, 97% sequence similarity was used as the OTU partition threshold, and a total of 626 OTUs were detected. Of these, the number of OTUs detected in the MN (normal group), MD (treatment group) and MM(model group) was 283, 230 and 443, respectively. From the Venn diagram (Fig 1), it can be seen that the number of unique OTUs in the MM group is surprisingly high, while that in the MN group and the MD group is only 36 and 16. The number of bacterial species in the model group was significantly higher than that in the treatment group and the normal group, while the number of bacterial species in t the latter two groups was similar.

**Fig 2:**
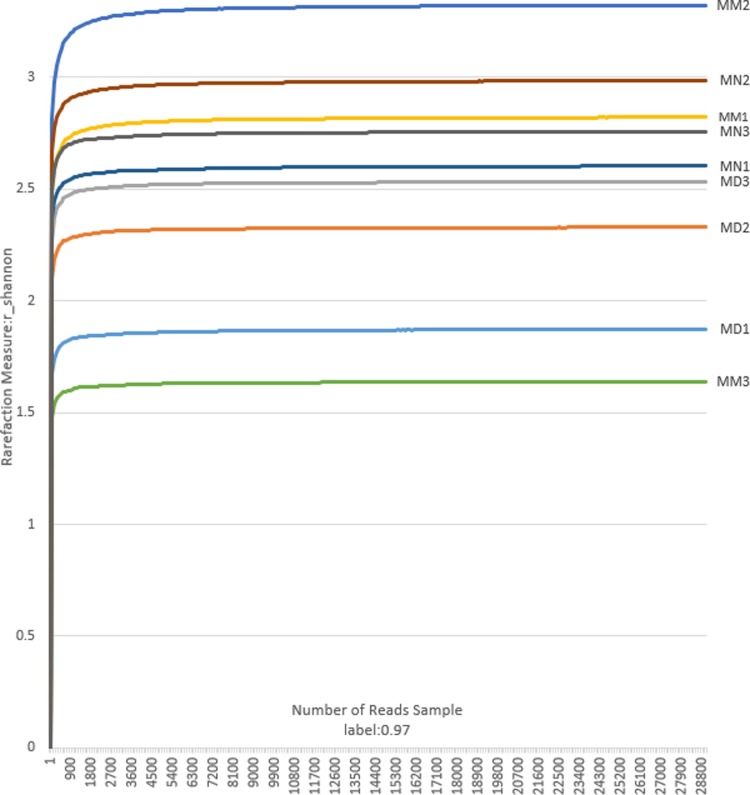
The Shannon index. The sparse analysis diagram of the Shannon index in the normal group (MN), the model group (MM) and the treatment group (MD).

**Fig 3:**
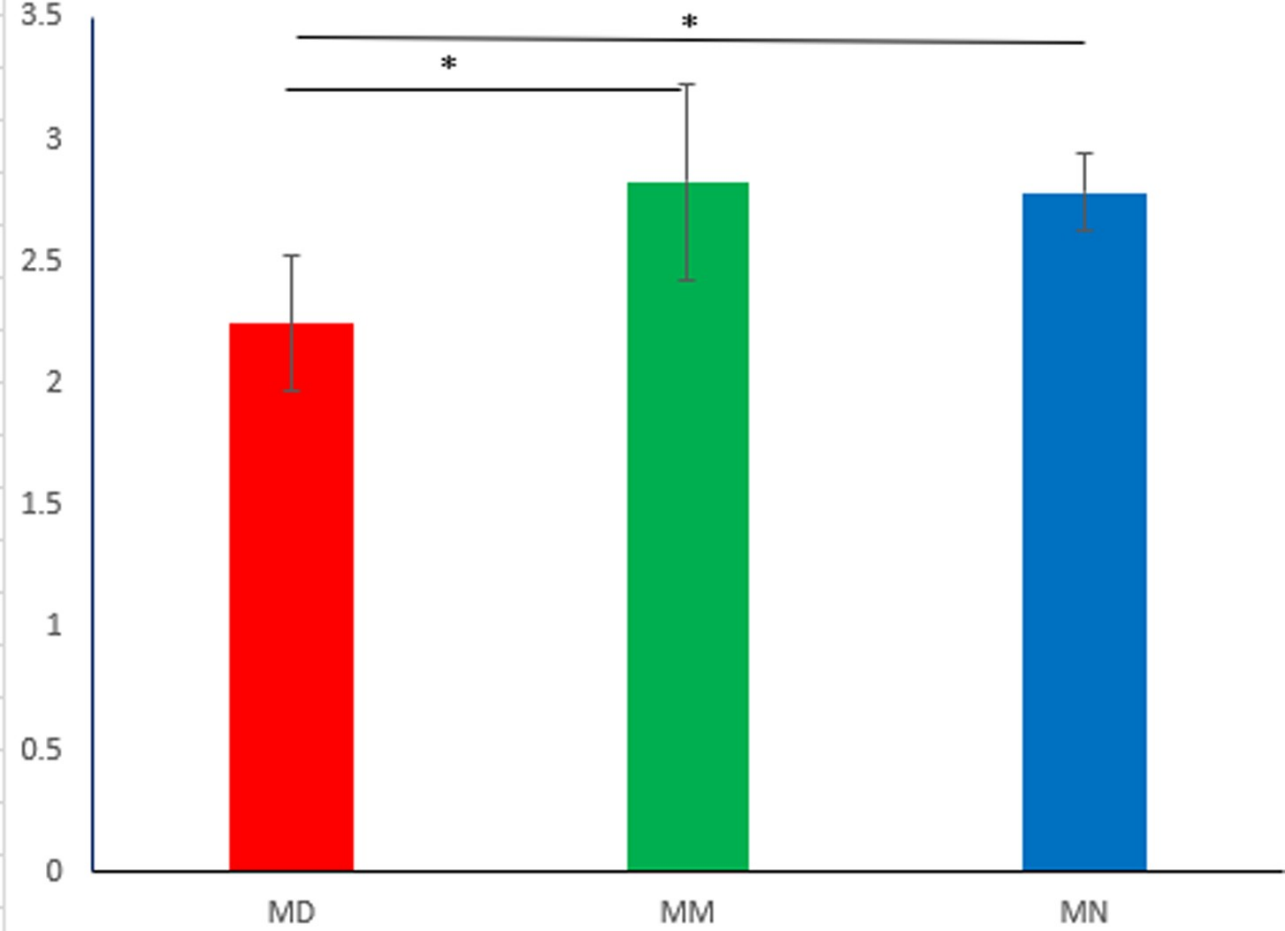
Alpha diversity analysis of the Shannon index in the normal group (MN), the model group (MM) and the treatment group (MD), *P<0.05.

The Shannon-Wiener curve was plotted based on the OTU information at the 97% similarity level and the diversity index at each sequencing depth, which exhibited the microbial diversity index in the intestinal mucosa. In addition, the curves constructed based on the microbial diversity of mucosal samples at different depths revealed whether the sequencing depth of mucosal samples is reasonable, and whether it can reflect the majority of microbial information in the samples. Fig 2 is the Shannon index analysis chart of 9 small intestinal mucosa samples. The curves of all stabilized samples showed an increase in sequencing volume, indicating that the sequencing depth was reasonable and revealed the majority of microbial information, which is the perquisite for the follow-up analyses. The Shannon diversity index considers synthetically the richness and uniformity of the community and takes into account the richness of microbial communities and rare OTUs. It can be seen from statistical analysis (Fig 3) that the Shannon index of the MN and MM group was significantly different from that of the MD group (*P*<0.05) The reason for this may be that treatment with high-dose antibiotics caused a decrease in the diversity of mucosal bacteria during the study period. Destruction of bacterial community structure in the MM group led to the proliferation of opportunistic bacteria during the period of spontaneous recovery, which was similar to secondary infection. From our perspective, the diversity of MM group which was similar with MN group was abnormal. This could be attribute to the subsequent response to high doses of antibiotics treatment, which could also explain why the unique OTUs of the MM group were unusually high, while *D*. *hansenii* inhibited the proliferation of opportunistic bacteria. It was difficult for the MD group to restore bacterial diversity in a short time. In general, the higher the diversity index, the more equitable the structure of the bacteria. However, it has been reported that a higher diversity index does not mean a higher level of bacteria in the body[16]. The reason for this may also be the excessive proliferation of opportunistic bacteria.

### Analysis of bacterial community structure

**Fig 4:**
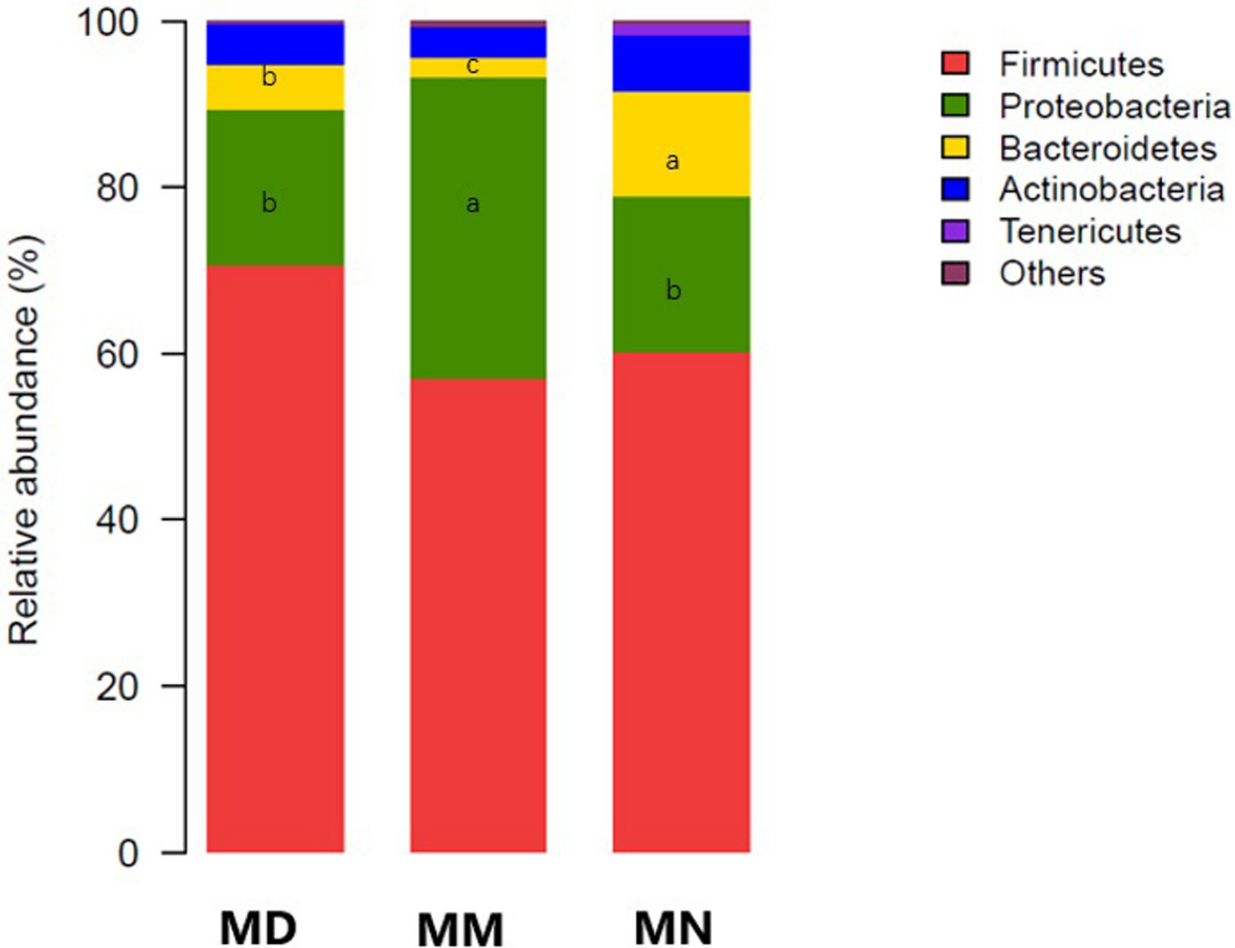
Bacterial distribution at the phylum level. Bacterial distribution based on the phylum taxonomical level in the normal group (MN), the model group (MM) and the treatment group (MD). Different lowercase letters indicated significant difference(*P*<0.05).

**Fig 5:**
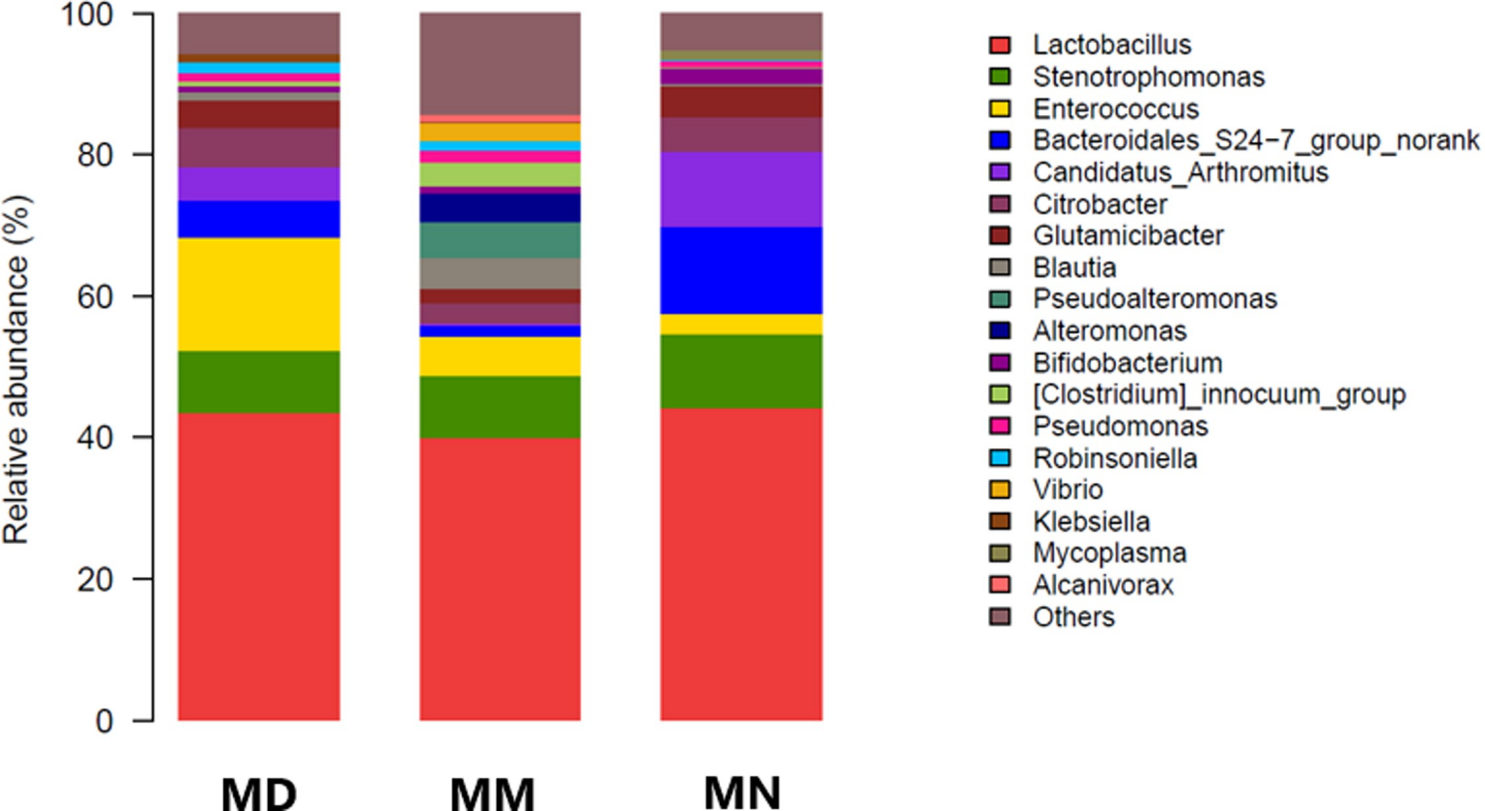
Bacterial distribution at the genus level. Bacterial distribution based on the genus taxonomical level in the normal group (MN), the model group (MM) and the treatment group (MD).

**Fig 6:**
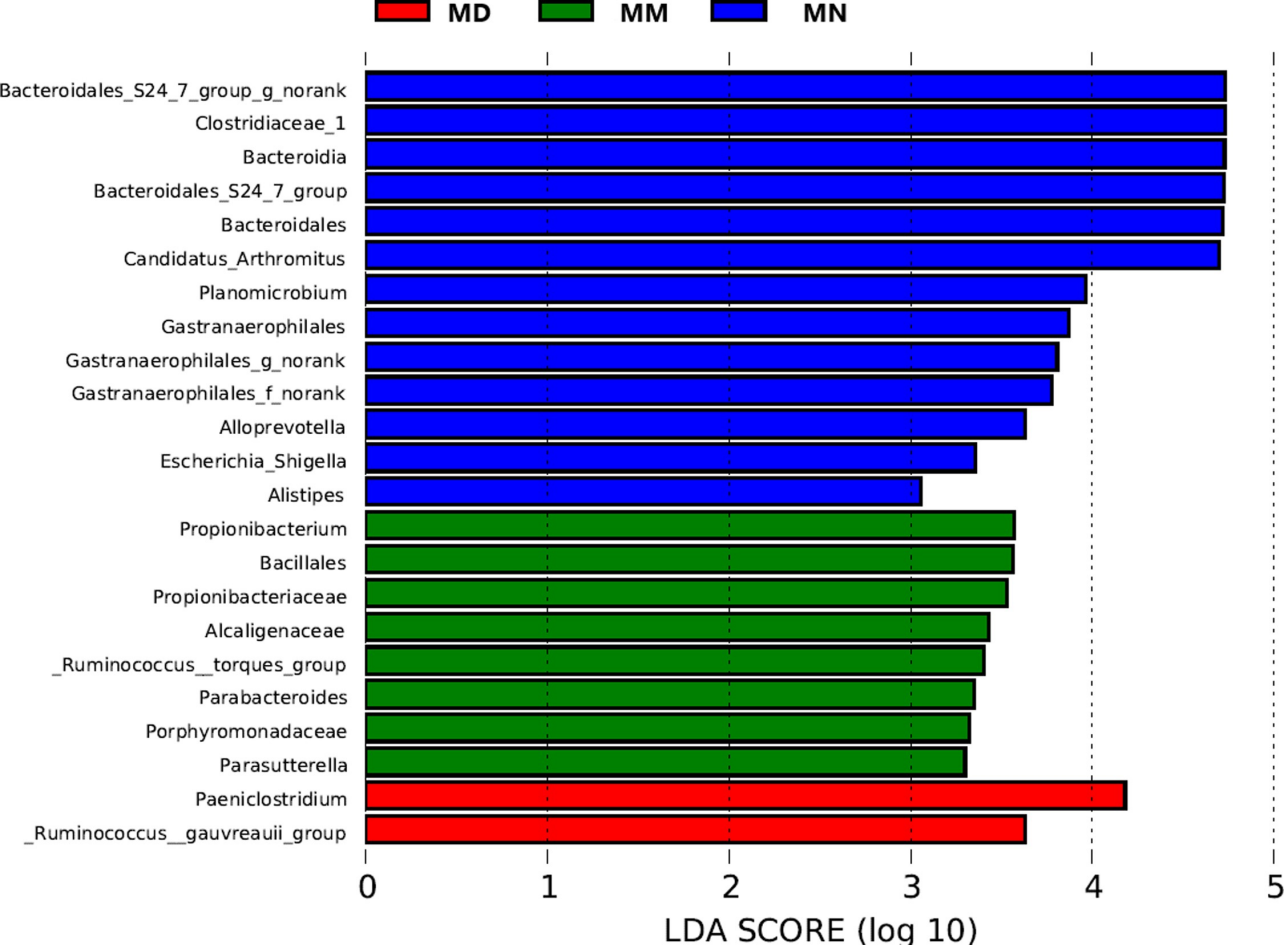
Histogram of the Linear discriminant analysis. Histogram of the Linear discriminant analysis (LDA) coupled with effect size measurements (LEfSe) identified the most differentially abundant genera between the MN, MM and MD groups.

Based on the OTU classification results, QIIME was used to obtain the composition and taxon distribution of mucosal samples in each group at the phylum level (Fig 4) and genus level (Fig 5), and the results were presented in a histogram. In addition, LEfSe analysis was carried out by submitting the relative abundance matrix at the genus level using the Galaxy online analysis platform, and the key colony members in each group were screened and presented in Fig 6.

Mouse intestinal mucosa mainly contained *Firmicutes*, *Proteobacteria*, *Bacteroidetes*, *Actinobacteria* and *Tenericutes* at the phylum level (Fig 4). After antibiotic treatment and *D*. *hansenii* treatment, the intestinal mucosa of mice in each group was still dominated by *Firmicutes*, while the abundance of *Proteobacteria* was different, with a relative abundance in the normal group, the model group and the treatment group of 18.78%, 36.12% and 18.77%, respectively. The abundance in the model group was obviously higher, while the abundance in the treatment group and the normal group remained at the same level. Differences were also observed in *Bacteroides*. The abundance of *Bacteroides* in the treatment group and the model group was significantly lower than that in the normal group (*P*<0.05).

A total of 280 taxa in the intestinal mucosa were identified at the genus level (Fig 5). The dominant bacteria in the intestinal mucosa of each group were *Lactobacillus*, indicating that *Lactobacillus* has adhesive ability and is very stable. Of the remaining 17 genera with high abundance, *Stenotrophomonas*, *Enterococcus*, *Bacteroidales S24-7 group norank*, *Candidatus Arthromitus*, etc. *Pseudoalteromonas*, *Alteromonas*, *Vibrio* and *Alcanivorax* in the model group all exhibited relatively high abundance, and all four genera belong to *Proteobacteria*, and this corresponded to the difference in *Proteobacteria* at the phylum level. It also confirmed that the model group had a higher number of OTUs in the Venn diagram. Therefore, the above findings indicate that antibiotic treatment can destroy the intestinal mucosal microbial ecosystem and promote the proliferation of opportunistic bacteria, while treatment with *D*. *hansenii* can effectively inhibit the proliferation of opportunistic bacteria and maintain the intestinal microbial ecosystem. It is worth mentioning that *Akkermansia*, a probiotic in the human intestinal tract, is also found in the mucosa of normal mice, however its abundance was low, and was not detected in the model group and the treatment group. This indicated that *Akkermansia* was very sensitive to antibiotics.

LEfSe is an analytical method based on LDA of effect size, which can screen out the key colony members in each group[17,18].Data from Fig6 demonstrated that *Bacteroidia*, *Bacteroidales*, *Bacteroidales S247 group*, *Bacteroidales S24 groupnorank*, *Alloprevotella*, *Alistipes*, *Gastranaerophilales*, *Gastranaerophilales f norank*, *Clostridiaceae 1*, *Candidatus Arthromitus* and *Escherichia Shigella* (*P*<0.05) were prevalent bacteria in the normal group. These colony members may be antibiotic-sensitive bacteria which were decreased in the model group and the treatment group (*P*<0.05) after antibiotic treatment. *Bacillales*, *Porphyromonadaceae*, *Propionibacteriaceae*, *Alcaligenaceae*, *Planomicrobium*, *Propionibacterium*, *Parabacteroides* and *Ruminococcus torques group* (*P*<0.05) were the key colony members in the model group. These colony members may be both opportunistic proliferating bacteria and insensitive to antibiotics. In the treatment group, there were only 2 dominant bacteria which were *Paeniclostridium* and *Ruminococcus gauvreauii* group (*P*<0.05). From the above analysis, we concluded that antibiotic treatment can cause the decline of many bacterial microbiota, and treatment with *D*. *hansenii* effectively inhibits the proliferation of opportunistic bacteria.

### β-diversity analysis

**Fig 7:**
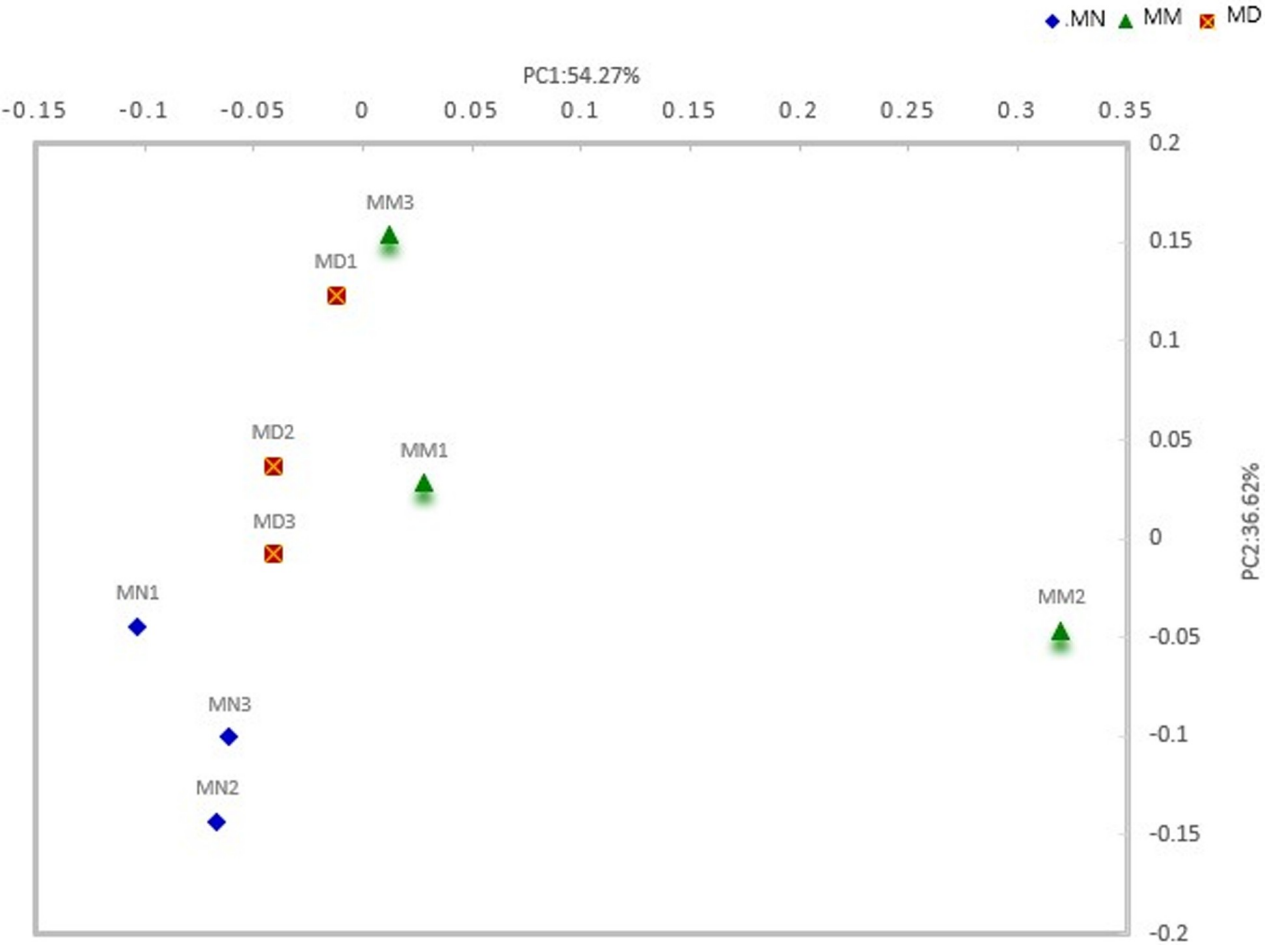
Composition of the microbial community. Sample 2d collation diagram analyzed by Weighted UniFrac PCoA.

There is a phylogenetic relationship between the microbial members of a community. UniFrac distance comprehensively reflects the degree of similarity among community samples by comparing the phylogenetic relationships between different communities with their unique OTUs[19]. As shown in Fig 7, mucosa samples from the treatment group, the model group and the normal group were significantly varied, indicating that there were certain differences in the composition of the microbial community between the samples. In the direction of PC1, the normal group and the treatment group were significantly different from the model group, and PC1 was the principal coordinate component of the maximum interpretation of data changes, indicating that the mucosal community structure composition of the normal group and the treatment group was similar, but significantly different from the model group. The distribution distance between the normal group and the treatment group was adequate, which could be well grouped.

## Discussion

In a previous study from our research group, the intestinal microbiota was disturbed and led to diarrhea following mixed antibiotic treatment[2], which is consistent with the results of the present study. The effect of *D*. *hansenii* on the microbial diversity of intestinal contents was studied in mice with dysbiosis[10]. The results showed that *D*. *hansenii* was able to adjust the diversity of intestinal lumen microbiota and restored the abundance of *Bacteroidaceae*[10]. In the present study, we aimed to identify further evidence of *D*. *hansenii* treatment in mice with dysbiosis diarrhea from the perspective of intestinal mucosa microbial diversity. According to the previous study on intestinal lumen microbiota[10], there were 1207 OTUs detected in the intestinal contents, and the number of OTUs in the model group, the treatment group and the normal group was 894, 849 and 849, respectively. These findings suggested that the number of bacterial species in the intestinal contents was significantly higher than that in the intestinal mucosa. These results also indicated that only a minority of bacterial species in the intestinal tract had mucosa adhesion ability, and most of the bacterial species were transient. According to the Shannon index curve obtained in this study, it is difficult to restore the diversity of intestinal microbiota in a short time through the treatment of *D*. *hansenii*, which in accordance with the previous study on intestinal microorganisms. In addition, this study confirmed that *D*. *hansenii* could control the proliferation of opportunistic bacteria in the mucosa of mice with an intestinal microbiota disorder and inhibit secondary infection caused by antibiotic treatment. From the PCoA results, *D*. *hansenii* contributes to the recovery of microbial community structure of dysbiosis mucosa to the normal-group level, while microbial community structure of the model group showed significantly different compared to the normal group. Based on the results of β diversity analysis in the previous study[10] microbiota structure on content in the normal group was clearly distinguished from that in the model group and the treatment group, which was different from the data obtained in this study. The reason for this may be that, on the basis of *D*. *hansenii*'s ability to treat antibiotic-induced diarrhea, adhesion of the mucosal microorganisms better demonstrated that *D*. *hansenii* could treat diarrhea by adjusting the intestinal microecology. Moreover, the difference in bacterial structure between the groups was analyzed at the phylum level and the genus level. Antibiotic treatment induced the proportion of *Proteobacteria*, and alleviated the proportion of *Bacteroidetes*, while *D*. *hansenii* treatment inhibited the increase in *Proteobacteria*. Compared with the analysis of bacterial distribution in the intestinal contents[10], the proportions of *Proteobacteria* and *Bacteroides* in the mucosa were different from those in the intestinal contents. The proportion of *Bacteroides*(41.5%) was significantly higher than that of *Proteobacteria*(2.10%) in the intestinal contents (analysis by He *et al*.), while the proportion of *Bacteroides* (12.61%) was significantly higher than that of *Proteobacteria* (18.78%) in the intestinal mucosal, which indicated that the adhesion ability of *Proteobacteria* was stronger than that of *Bacteroides*. In conclusion, *D*. *hansenii* slowly restored disordered mucosal microorganisms in the microbial community structure similar to the normal group, and inhibited the proliferation of opportunistic bacteria. In addition, high-dose antibiotic treatment can cause mucosal dysbiosis and the proliferation of opportunistic bacteria during the self-recovery period.

*Candidatus Arthromitus* is a spore-forming anaerobic bacterium that is adsorbed on the apical or lateral side of the animal's intestinal mucosa. It is closely related to the intestinal mucosal immunity of the host, which promotes immune maturation and enhances host resistance[20,21]. According to the LefSe analysis carried out in this study, *Candidatus Arthromitus* was only prevalent in the normal group, indicating that treatment with large doses of antibiotics had a marked influence on *Candidatus Arthromitus*, and recovery was difficult in a short time period after *D*. *hansenii* treatment. *Akkermansia muciniphila*[22,23] is associated with degradable mucoprotein which is implanted in the intestinal mucosa, and is negatively correlated with obesity, diabetes, inflammation and metabolic disorders, and accounts for 1% of the total amount of bacteria in the human intestinal tract[24]. However, this study showed that the proportion of *Akkermansia muciniphila* in the jejunal mucosa of mice was very low. The reason for this may be that *Akkermansia muciniphila* was mainly concentrated in the colonic mucosa (it was reported that the colonization rate of *Akkermansia muciniphila* in the colonic mucosa was significantly higher than that in the small intestine), or the number of *Akkermansia muciniphila* was low in the mucosa of the selected mice in this study, which requires further study. Generally speaking, we could draw a conclusion that treatment with *D*. *hansenii* could help the structure of the mucosal microbiota recover to a similar microbiota structure to the normal group, but could not help the microbiota diversity restore to the microbiota diversity to the normal group in a short treatment period. At the same time, treatment with *D*. *hansenii* could effectively inhibit the proliferation of opportunistic bacteria such as *Pseudoalteromonas*, *Alteromonas*, *Vibrio*.

## References

[1] Doron SI, Hibberd PL, Gorbach SL. Probiotics for prevention of antibiotic-associated diarrhea. J Clin Gastroenterol, 2008;42 (S2): 58–63. 10.1097/MCG.0b013e3181618ab7 18542041

[2] Long CX, Lu He, YF Guo, YW Liu, NQ Xiao, ZJ Tan. Diversity of bacterial lactase genes in intestinal contents of mice with antibiotics-induced diarrhea. World J Gastroenterol,2017;23(42):7584–7593 10.3748/wjg.v23.i42.7584 29204058 PMC5698251

[3] Jiang XL, Cui HF. An analysis of 10218 ulcerative colitis cases in China. World J Gastroenterol,2012;8(1):158–161. 10.3748/wjg.v8.i1.158 PMC4656610 11833094

[4] Eamonn MD, Hussein MD, Barbara MD, Bhatia MD, Boeckxstaens MD, De Giorgio MD, et al A global perspective on irritable bowel syndrome: A consensus statement of the world gastroenterology organization summit task force on irritable bowel syndrome. J Clin Gastroenterol,2012;46(5):356–366. 10.1097/MCG.0b013e318247157c 22499071

[5] Iradj S, Julien T, Françoise RT, Jean P. Roperch, L Sophie. Microbial dysbiosis in colorectal cancer (CRC) patients. PloS one,2011, 6(1):16393–16397. 10.1371/journal.pone.0016393 PMC3029306 21297998

[6] Belkaid Y, Hand TW. Role of the microbiota in immunity and inflammation. Cell. 2014,157(1):121–141. 10.1016/j.cell.2014.03.011 24679531 PMC4056765

[7] Rooks MG, Garrett WS. Gut microbiota, metabolites and host immunity. Nat Rev Immunol. 2016;16: 341–52. 10.1038/nri.2016.42 27231050 PMC5541232

[8] Marteau P, Lepage P, Mangin I, Suau A, Dorã J, Pochart P. Review article: gut flora and inflammatory bowel disease. Aliment Pharmacol Ther. 2004;20(Suppl 4):18–23. 10.1111/j.1365-2036.2004.02062.x 15352889

[9] Liu YJ, Xiao XY, Deng YL, Guo KX, She Y, Tan ZJ. Effects of Qiweibaizhusan combined with yeast on intestinal Lactobacillus diversity in diarrhea mice. Space Med Med Eng. 2016;29(3):175–180.

[10] He L, Long CX, Liu YJ, Guo YF, Xiao NQ, Tan ZJ. Effects of *Debaryomyces hansenii* treatment on intestinal microorganisms in mice with antibiotics-induced diarrhea. 3 Bitotech, 2017;7(5):347. 10.1007/s13205-017-0953-9 PMC5612875 28955644

[11] Long CX, Liu YW, He L, Tan QQ, Yu ZZ, Xiao NQ, et al Bacterial lactase genes diversity in intestinal mucosa of mice with dysbacteriosis diarrhea induced by antibiotics. 3 Biotech. 2018; 8:176 10.1007/s13205-018-1191-5 29556430 PMC5847641

[12] Magoc T, and Salzberg SL. FLASH: fast length adjustment of short reads to improve genome assemblies. Bioinformatics. 2011; 27:2957–2963. 10.1093/bioinformatics/btr507 21903629 PMC3198573

[13] Edgar RC. Search and clustering orders of magnitude faster than BLAST. Bioinformatics. 2010; 26:2460–2461. 10.1093/bioinformatics/btq461 20709691

[14] Segata N, Izard J, Waldron L, Gevers D, Miropolsky L, Garrett WS. Metagenomic biomarker discovery and explanation. Genome Biol. 2011,12. 10.1186/gb-2011-12-6-r60 PMC3218848 21702898

[15] Ramette A. Multivariate analyses in microbial ecology. FEMS Microbiol Ecol.2007; 62:142–160. 10.1111/j.1574-6941.2007.00375.x 17892477 PMC2121141

[16] Garg M, Hendy P, Ding JN, Shaw S, Hold G, Hart A. The effect of vitamin D on intestinal inflammation and fecal microbiota in patients with ulcerative colitis. J Crohns Colitis, 2018; 12(8):963–972. 10.1093/ecco-jcc/jjy052 29726893

[17] Wang SM, Chen L, He MZ, Shen JD, Li GQ, Tao ZR. Different rearing conditions alter gut microbiota composition and host physiology in Shaoxing ducks. Sci Rep. 2018; 8:7387 10.1038/s41598-018-25760-7 29743727 PMC5943461

[18] Zhang C, Chen L, He MZ, Shen JD, Li GQ, Tao ZR. Structural modulation of gut microbiota in life-long calorie-restricted mice. Nat Comuni. 2013; 4:2163. 10.1038/ncomms3163 PMC3717500 23860099

[19] Lozupone CA, Hamady M, Kelley ST, Knight R. Quantitative and qualitative beta diversity measure lead to different insights into factors that structure microbial communities. Appl Environ Microbiol. 2007; 73:1576–1585. 10.1128/AEM.01996-06 17220268 PMC1828774

[20] Yoshiyuki G, Casandra P, Gaku N, Cebula A, Carolyn L, Marta G D, et al Ivanov. Segmented filamentous bacteria antigens presented by intestinal dendritic cells drive mucosal Th17 cell differentiation. Immun. 2014;40(4): 594–607. 10.1016/j.immuni.2014.03.005 PMC4084624 24684957

[21] Thompson CL, Mikaelyan A, Brune A. Immune-modulating gut symbionts are not “Candidatus Arthromitus”. Mucosal Immun. 2013; 6:200–201. 10.1038/mi.2012.91 23013646

[22] Dao MC, Everard A, Aron-Wisnewsky J, Sokolovska N, Prifti E, Verger EO, et al Akkermansia muciniphila and improved metabolic health during a dietary intervention in obesity: relationship with gut microbiome richness and ecology. Gut, 2016;65(3):426–436. 10.1136/gutjnl-2014-308778 26100928

[23] Le Chatelier E, Nielsen T, Qin J, Prifti E, et al Richness of human gut microbiome correlates with metabolic markers. Nat. 2013;500(7464):541–546. 10.1038/nature12506 23985870

[24] Derrien M, Collado MC, Ben-Amor K. The Mucin degrader Akkermansia mciniphila is an abundant resident of the human intestinal tract. Appl Environ Microbiol. 2008;74(5):1646–1648. 10.1128/AEM.01226-07 18083887 PMC2258631

